# Uganda National Institute of Public Health: Establishment and Experiences, 2013–2021

**DOI:** 10.9745/GHSP-D-21-00784

**Published:** 2022-08-30

**Authors:** Alex Riolexus Ario, Issa Makumbi, Daniel Kadobera, Lilian Bulage, Felix Ocom, Benon Kwesiga, Dennis F. Jarvis, Sandra Nabatanzi, Jaco Homsy, Flora Banage, Vance Brown, Julie R. Harris, Amy L. Boore, Lisa J. Nelson, Sue Binder, Henry G. Mwebesa, Jane R. Aceng

**Affiliations:** a Uganda National Institute of Public Health, Kampala, Uganda.; b Ministry of Health, Kampala, Uganda.; c U.S. Centers for Disease Control and Prevention, Atlanta, GA, USA.; d U.S. Centers for Disease Control and Prevention, Kampala, Uganda.; e International Association of National Public Health Institutes, Global Health Institute, Emory University, Atlanta, GA, USA.

## Abstract

Uganda is an ecological hot spot with porous borders that lies in several infectious disease transmission belts, making it prone to disease outbreaks. To prepare and respond to these public health threats and emergencies in a coordinated manner, Uganda established the Uganda National Institute of Public Health (UNIPH) in 2013.

Using a step-by-step process, Uganda’s Ministry of Health (MOH) crafted a strategy with a vision, mission, goal, and strategic objectives, and identified value additions and key enablers for success. A regulatory impact assessment was then conducted to inform the drafting of principles of the bill for legislation on the Institute.

Despite not yet attaining legal status, the UNIPH has already achieved faster, smarter, and more efficient and effective prevention, detection, and response to public health emergencies. Successes include a more coordinated multisectoral, disciplined, and organized response to emergencies; appropriate, timely, and complete information receipt and sharing; a functional national lab sample and results transportation network that has enabled detection and confirmation of public health events within 48 hours of alert; appropriate response to a confirmed public health event in 24–48 hours; and real-time surveillance of endemic- and epidemic-prone diseases.

In this article, we document success stories, lessons learned, and challenges encountered during the unique staged process used to develop the components of the UNIPH. The creation of an integrated disease control center has proven to yield better collaboration and synergies between different arms of epidemic preparedness and response.

## Abstract (precis)

Since 2013, the Uganda National Institute of Public Health (UNIPH) has successfully collaborated with partners and secured donor funding as it works toward legal establishment as an autonomous entity eligible for government funding. Countries in Africa and beyond can learn from the process Uganda undertook to develop the UNIPH.

## Key Messages

- Building the Uganda National Institute of Public Health (UNIPH) in stages, with collaboration between the Ministry of Health and other partners for financial and technical support, ensured a solid establishment of its key structures.
- As Uganda consolidates its public health functions by establishing the UNIPH through an Act of Parliament, smarter, faster, and more effective prevention, detection, and response to public health emergencies is already occurring in the country.

## INTRODUCTION

National public health institutes (NPHIs) serve as focal points of countries’ efforts to protect and improve the health of their citizens. Working with multisectoral government agencies at national and subnational levels, they provide evidence-based leadership and public health services and help catalyze responses of countries to important public health challenges, including global health security. NPHIs are multifaceted organizations that include groups of individuals with a wide range of skills and experience who perform critical public health functions, such as surveillance, research, prevention, preparedness, and emergency management and response. Because they are science based, NPHIs are a trusted source of information and evidence for policy and decision makers. In most cases, NPHIs are part of the government, usually under or closely attached to the Ministry of Health (MOH).^1^

The nature and structure of NPHIs vary greatly between countries. Typically, they have been created to respond to specific problems, often related to infectious diseases, and have had programs added in response to new threats, interests of leadership, political considerations, efficiencies achieved by combining organizations, and other factors.^2^ Only lately have comprehensive conceptual frameworks, like the ones developed by the International Association of National Public Health Institutes (IANPHI) and the Africa Centers for Disease Control and Prevention (Africa CDC), been used to guide the formation or growth of NPHIs.^1^^,^^3^

When the Africa CDC was founded in 2017, it prioritized the development of NPHIs in member countries. At its inception, Africa CDC noted that functional NPHIs are critical for effective outbreak response, guiding health policies and strategies through science and data, building health workforce capacity, accelerating implementation of the International Health Regulations (IHR), and building robust disease surveillance systems.^1^^,^^4^ Indeed, the core vision underlying NPHIs is that success in building strong health systems in Africa requires member states to have strong public health institutions. The IANPHI has worked collaboratively with countries, especially those categorized as low resource, to strengthen existing NPHIs or set up new ones. By December 2021, only 14 African countries had had their NPHIs fully established by a statutory instrument: Algeria, Angola, Burkina Faso, Cape Verde, Ethiopia, Guinea Bissau, Liberia, Kenya, Malawi, Mozambique, Nigeria, Somalia, Tunisia, and Zambia. However, many countries including Botswana, Namibia, Rwanda, South Africa, South Sudan, and Uganda are on the path to fully establishing their NPHIs with support from U.S. Centers for Disease Control and Prevention (CDC), IANPHI, Africa CDC, and other partners.^3^^,^^5^

From its onset, the Uganda National Institute of Public Health (UNIPH) benefited from the IANPHI framework for developing NPHIs. UNIPH operates as a closely linked network of key organizations for carrying out core public health functions in Uganda. In this article, we describe the processes followed in setting up the UNIPH and the successes and challenges encountered on the journey of its development.

## DEVELOPING THE UNIPH

The set-up process commenced with the MOH’s vision to develop UNIPH. To ensure the legal and sustainable status of the UNIPH, its development was defined by a process of peer-to-peer learning; participatory planning meetings facilitated by IANPHI, U.S. CDC, RESOLVE to Save Lives, African Field Epidemiology Network, and Management Sciences for Health; task force formation; stakeholder consensus meetings; political leadership and support; white paper development; use of the IANPHI Staged Development Tool; regulatory impact assessment (RIA); and principles for drafting of the legal instrument for UNIPH.

### MOH Vision for UNIPH Development

The move toward a more coordinated approach to prevention, detection, and response to public health emergencies was conceived by the MOH in early 2013. This followed several years of multiple major disease outbreaks, to which the response was deemed ineffective and uncoordinated among many technical players and donors. Examples include Uganda’s largest documented Ebola virus disease (EVD) outbreak, which occurred in 2000–2001 in Gulu District, registering 425 cases and 224 deaths, and another EVD outbreak in 2007 in Bundibugyo, registering 116 cases and 39 deaths, including many health workers.^6^ This was compounded by the low in-country technical capacity to respond to the country’s public health needs.^4^

Following several major disease outbreaks, the Uganda MOH sought a more coordinated approach to public health emergency response.

### UNIPH Task Force Creation

In May 2013, a team of MOH officers led by the Director General of Health Services visited the CDC in Atlanta, United States, to learn how to develop an NPHI. Representatives of IANPHI also participated in the visit. Following the event, a task force of 21 members was formed with specific terms of reference to kick-start the development of the Institute. The composition of the task force reflected members’ expertise in laboratory systems, disease control programs, regional networks, epidemiology and surveillance, workforce development, health information systems, health policy, and research. Members were selected from the following organizations: MOH (Departments of National Disease Control; Planning; Communications; Health Promotion; Disease Prevention and Care Programs; Health Information; Laboratory; Epidemiology and Surveillance; and Human Resource Development), the World Health Organization’s Uganda Office (WHO-Uganda), the U.S. and Uganda CDC Offices, Makerere University Kampala (College of Veterinary Medicine and College of Health Sciences), Uganda Wildlife Authority, UNICEF, and Uganda Virus Research Institute. A core team of 5 members from the task force attended a Leadership and Management Institute training in Atlanta, United States in September 2013 and submitted a project proposal for developing the UNIPH. In December 2013, a team from IANPHI and CDC’s NPHI program made an engagement visit to Uganda and held discussions with the UNIPH Task Force, the MOH leadership, and WHO-Uganda.

At various points throughout the development process, the task force conducted several consultative meetings to solicit technical input to inform the UNIPH White Paper and RIA from CDC Atlanta, IANPHI, Policy Analysis Unit, MOH, and Cabinet Secretariat throughout the process.

Political leadership supported the establishment of the UNIPH from the start. In June 2013, the Minister of Health wrote a letter to the U.S. CDC Director expressing a need for support to develop the UNIPH. The CDC Director accepted and came to Uganda to officiate a groundbreaking ceremony of the National Health Laboratory Services complex at the proposed UNIPH campus.

Political leadership in Uganda supported the establishment of the UNIPH from the start.

### Implementation Phases of UNIPH

**FIGURE 1:**
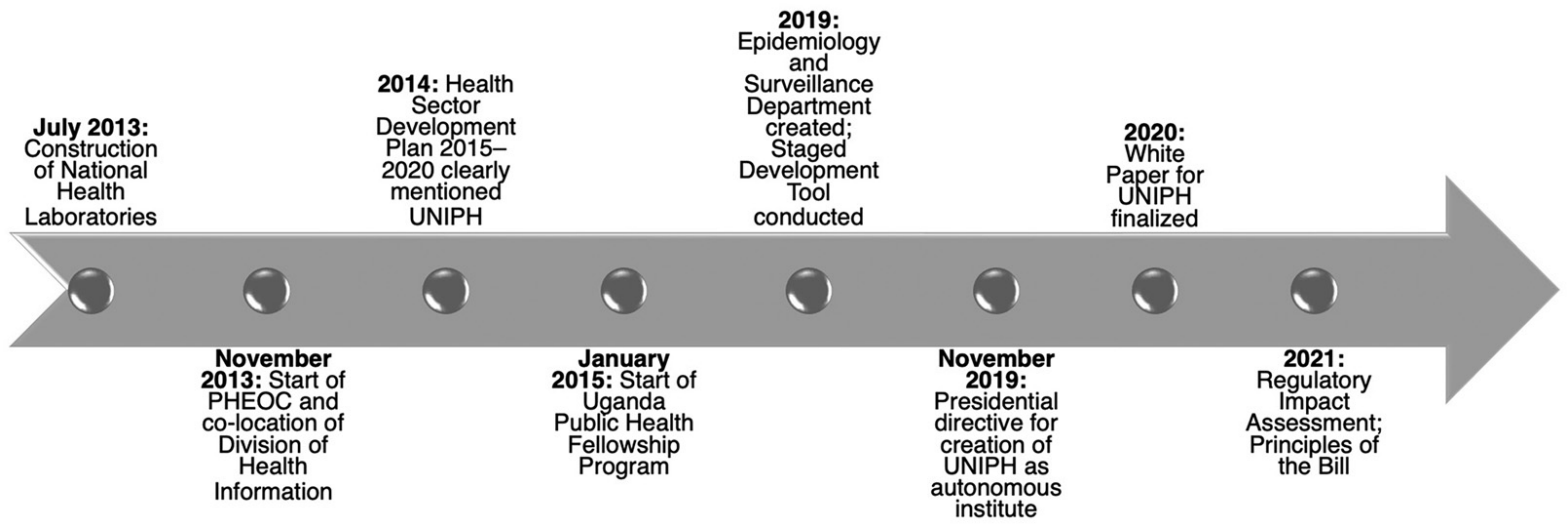
Implementation Phases of the Uganda National Institute of Public Health, 2021 Abbreviations: HSDP, Health Sector Development Plan; PHEOC, Public Health Emergency Operations Centre; UNIPH, Uganda National Institute of Public Health.

1. July 2013: Construction of the National Health Laboratories began at the main campus after the groundbreaking ceremony overseen by the U.S. CDC Director and the Minister of Health (Figure 1).
2. November 2013: Colocation of the PHEOC and the MOH Division of Health Information near the MOH Headquarters in Kampala.
3. 2014: Finalization of the Health Sector Development Plan 2015–2020, which made clear mention of the UNIPH.
4. January 2015: the Uganda Public Health Fellowship Program commenced and colocated with the PHEOC, MOH Division of Health Information, and MOH Department of Epidemiology and Surveillance. The Division of Health Information and the Epidemiology and Surveillance Department currently functioning within MOH will form the National Health Information Services and Disease Epidemiology and Surveillance components of the Institute.
5. September 2015: The Minister of Health gave full support to the development of the UNIPH and directed the task force to complete the strategy and draft a cabinet memorandum for preparation of the UNIPH Bill. Uganda’s Minister of Health was the Director General of Health Services at the time of the conceptualization of the UNIPH and has continued to endorse and champion its development.
6. November 2016: National Health Laboratories officially launched by the President of Uganda. It is functional with staff formally working under the Central Public Health Laboratories.

### White Paper Development

Several ensuing UNIPH Task Force meetings led to the development of the Strategy for UNIPH, which was later changed to the White Paper for UNIPH. The White Paper was informed by a situation analysis of the many policy and strategic documents of the MOH,^7^ and incorporated a justification, added value of UNIPH, and implementation plans. The White Paper identified the following essential public health functions in need of capacity building or strengthening: research (evidence base for policies and programs), health education and promotion, public health workforce development, population health assessment, and disease surveillance and response. Based on these public health functions, key components of the Institute were identified that in turn informed the structure and directorates of the UNIPH. Following discussion of this guiding document at a stakeholders’ meeting in Kampala, consensus was reached on the formation of the UNIPH in June 2016.

### Staged Development Tool

The IANPHI created the Staged Development Tool to help NPHIs assess their current capacity and develop roadmaps for achieving a higher level of functionality. The focal person for development of the UNIPH attended a workshop on the Staged Development Tool and used it to improve the development process.^8^ In a 3-step sequence of assessing gaps, prioritizing, and planning, we established the baseline capacity using the discussion guide focusing on the 28 Staged Development Tool technical areas, determined maturity in priority areas, and identified areas for improvement. This informed the strategic planning and improved the efficiency and effectiveness of implementing the development process of the Institute.

The IANPHI Staged Development Tool was used to inform strategic planning and improve efficiency and effectiveness during the development process.

### RIA

With support from the Policy Analysis Unit at MOH, the Department of Policy Development and Capacity Building, Office of the President (Cabinet Secretariat), and development partners, the RIA preparation process commenced in September 2021. The RIA was conducted to:

- Identify the policies and laws that were in effect guiding public health undertakings in the country
- Provide a comprehensive definition of public health
- List all the issues challenging the country in its efforts to address public health needs and therefore the need for the establishment of an NPHI
- Identify causes of the challenges and their effects on the health system

After consultations were conducted and the final report produced, the RIA informed the drafting of the Principles of the Bill (cabinet memorandum).

## ESTABLISHING THE UNIPH

### Vision, Goal, and Objectives of UNIPH

The sustained effort of the Task Force, MOH, and partners ensured that the country steadily moved toward actualizing the vision of establishing the UNIPH (Box 1).

BOX 1 **Vision, Mission, Goal, and Objectives of the Uganda National Institute of Public Health**

**Vision:** to contribute toward a healthy and productive population.

**Mission:** to provide leadership in disease surveillance, prevention, and control, and innovation in multidisciplinary research to generate evidence to inform policies and practices in public health and state-of-the-art training of human resources for health.

**Goal:** to provide strategic direction to improve public health based on sound scientific evidence and best available practice to contribute to attainment of a good standard of health for all people in Uganda.

**Strategic objectives:**

- Develop broad and deep data collection abilities across the country
- Integrate critical disease control operations
- Promptly detect and respond to public health events
- Provide leadership for identification and implementation of research that is responsive to public health issues
- Develop a robust and sustainable national public health reference laboratory capacity to address national public health concerns
- Build capacity in surveillance, response, control, prevention, and research
- Establish systems for tracking and monitoring response to public health functions
- Develop effective and efficient health communication services
- Sustainably manage disease control activities

### Components and Funding of UNIPH

A stakeholder consensus meeting yielded the following recommendations regarding the components and funding of UNIPH.

- Create 8 directorates: Disease Epidemiology and Surveillance, Public Health Emergency Operations Centre (PHEOC), Zoonotic, Environmental and Emerging Health Events, National Health Information Services, National Health Laboratory Services, Workforce Development, Public Health Research, and Finance and Administration.
- Appoint directors over these who will report to the Executive Director. The Executive Director will be the overall head of UNIPH and answerable to a Board of Directors nominated by the Minister of Health and appointed by the President of the Republic of Uganda.
- Legally establish UNIPH as an autonomous, self-sustaining government parastatal. Securing this status will automatically generate a dedicated line in the annual government budget with specific allocations to UNIPH. Once autonomous, the Institute can qualify to solicit for and manage funds independently through grant applications from government and nongovernmental institutions, including academia, nongovernmental organizations, and philanthropies in the public and private sectors.
- Organize UNIPH to function through affiliations and memoranda of understanding with recognized institutions, based on their functions and interests in response to public health needs in the country. The following institutions/ministries were identified as key players: MOH; Ministry of Agriculture, Animal Industry and Fisheries; Ministry of Water and Environment; National Environment Management Authority; Uganda Wildlife Authority; Uganda Virus Research Institute; Infectious Diseases Institute; Uganda National Health Research Organization; Mildmay Uganda; Makerere University Walter Reed Project; Makerere University-John Hopkins University Research Collaboration; Makerere University and other public universities.
- Strengthen UNIPH's leadership throughout its departments; clearly define the roles of every player from the Board of Directors to the operational level staff; appreciate the multisectoral and multidimensional nature of the Institute; ensure the prompt endorsement of required institutional arrangements and actions at the highest level; develop a clear strategic plan; and ensure good financial management and accountability in program implementation.

### National Coordination Mechanisms for Public Health Emergencies

**FIGURE 2:**
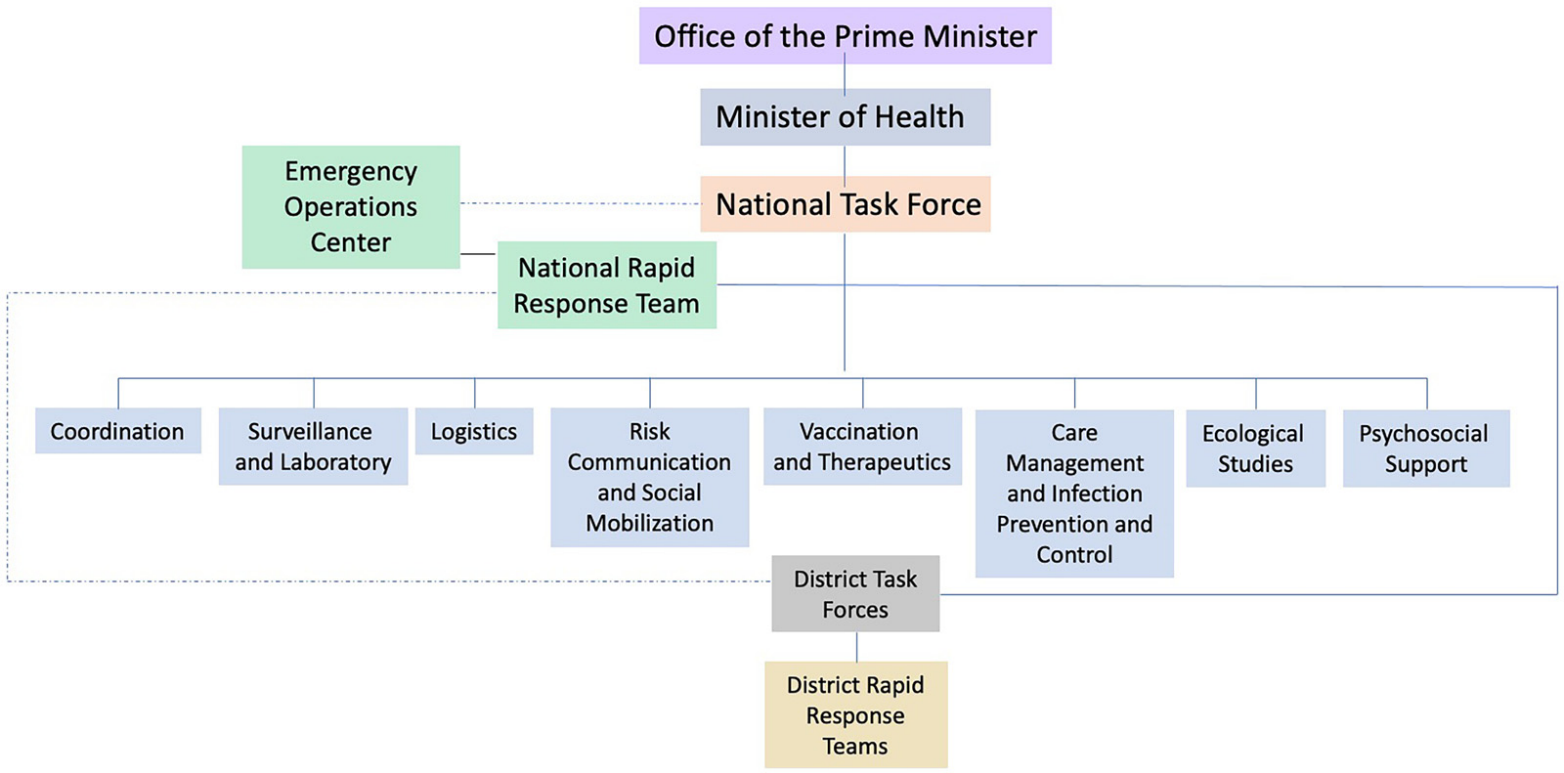
National Response Structure for Public Health Emergencies in Uganda

Creation of the UNIPH triggered the establishment of the national coordination mechanisms: National Task Force and its subcommittees, PHEOC, and District Task Forces (Figure 2). These mechanisms in Uganda are the parties responsible for executing the national emergency preparedness and response plan.

Creation of the UNIPH triggered establishment of the national coordination mechanisms, which are responsible for executing the national emergency plan.

The National Task Force subcommittees are: Coordination, Surveillance, and Laboratory; Case Management and Infection Prevention and Control; Risk Communication and Social Mobilization; Logistics; Vaccination and Therapeutics; Psychosocial Support; and Ecological Studies.

When a disease outbreak occurs, depending on the magnitude, the Director General of Health Services activates the national coordination mechanisms to mobilize the national resources (financial, human, materials) necessary for emergency management. When the national coordination mechanisms are not activated, staff assigned to these mechanisms perform their regular duties at their stations/offices. PHEOC staff are permanent and concentrate on normal preparedness activities, including training of response teams, drafting and revising response guidelines and standard operating procedures, and conducting corrective actions derived from previous emergency after-action reports. The National Task Force is multidisciplinary and includes officers appointed from different ministries, agencies, and partners. Subcommittee members are subject matter experts selected from relevant institutions and ministries across the country. The National Rapid Response Team consists of epidemiologists and other technical staff including clinicians and laboratorians who are assembled for the purpose of investigating and responding to an outbreak. The District Task Forces are multidisciplinary and at district level, while the District Rapid Response Team comprises technical staff operating as rapid responders at the district level.

In November 2018, the Minister of Health wrote a letter to the President of the Republic of Uganda with a clear justification for the need to establish UNIPH as an autonomous institution by an Act of Parliament. The President issued a directive on November 19, 2019, granting the creation of UNIPH as an autonomous institute by a statutory instrument.

As a legal autonomous institute, the UNIPH will be able to receive both government funds and funding from donor agencies.

The major changes that the legal instrument will make regarding the UNIPH are: (1) providing a legal status to the Institute, qualifying it to enter into the Mid-Term Expenditure Framework of the Government of Uganda; (2) streamlining the components of the Institute into 1 organogram with clear reporting channels and 1 leadership with executive authority; and (3) enabling the Institute to qualify as a "trusted institution" to receive funding from any donor agency. In this way, the UNIPH will qualify for appropriation of funds as per the annual budgeting process and have its own grant management system.

## ACHIEVEMENTS AND BENEFITS OF UNIPH

The UNIPH has enabled the country to register numerous successes in implementing the Global Health Security Agenda and scaling up IHR capacities to prevent, detect, and respond to public health events (Box 2).

- Promoted effective collaboration of all arms of the UNIPH, which has strengthened epidemic preparedness and response
- Coordinated and organized multisectoral responses to public health emergencies
- Fostered appropriate receipt and sharing of health security information
- Enabled timely detection, confirmation, and response to public health emergencies within 48 hours of receiving an alert because of a national network of community-, event-, and indicator-based epidemiological surveillance systems facilitated by an effective National Rapid Response Team, a robust lab testing capacity, and an efficient sample referral and transportation network
- Provided real-time surveillance of endemic- and epidemic-prone diseases
- Ensured control of most disease outbreaks at their source
- Used evidence-based policy decisions by the MOH and the National Task Force for Public Health Emergencies^9^

BOX 2 **Uganda National Institute of Public Health’s Value Added**

Uganda National Institute of Public Health (UNIPH) is the implementation arm of the Ministry of Health to prevent, detect, prepare for, and respond to public health emergencies. UNIPH has added significant value to Uganda’s capacity for disease prevention and control and public health response in the following technical areas:

- Enhanced surveillance systems
- Improved community preparedness and alerts
- Coordinated approach to public health emergencies
- Prompt availability and sharing of critical epidemiological information
- Improved communication with the leadership at various levels (relevant ministries, regions, districts, and the public)
- Improved emergency activation mechanisms
- Enhanced countermeasures to diseases of public health importance
- Improved public health outcomes and population health security
- Skilled and capable health workforce
- Cost savings across government sectors through shared physical and human resources

In summary, there is faster, smarter, more efficient and effective prevention, detection, and response to public health emergencies. This was evidenced by the 2017 Joint External Evaluation of Uganda’s IHR Core Capacities compared to the 2015 figures and continues to be true.^10^

The UNIPH has enabled faster, smarter, more efficient and effective prevention, detection, and response to public health emergencies.

### COVID-19 Response

The emergence of coronavirus disease (COVID-19) tested the response system in the country. By the time the country recorded its first case, Uganda was in preparedness mode for an EVD outbreak that had begun in the neighboring Democratic Republic of Congo. We immediately transitioned to COVID-19 preparedness and eventual response. The national coordination mechanisms were activated and an incident management structure was immediately put in place to manage the response as directed by the National Task Force for Public Health Emergencies. We managed to contain COVID-19 for 6 months before community transmission was registered.

The UNIPH houses the COVID-19 strategic room. The PHEOC has effectively and efficiently coordinated the response to the pandemic through development of strategic plans and partner and resource mobilization and coordination. The Uganda Public Health Fellowship Program has conducted multiple high-impact studies that have informed guidance and response to COVID-19 in the country. The program has supported and continues to support the national response to COVID-19 through conducting rapid assessments, epidemiological investigations, seroprevalence surveys, intra-action review, and promotion of vaccination uptake, among other activities. Sample collection, testing, and genomic sequencing is done in country by the National Health Laboratory Services and the Uganda Virus Research Institute. Since the declaration of the COVID-19 pandemic in March 2020, the Institute has also responded to yellow fever, Crimean-Congo hemorrhagic fever, measles, suspected food poisoning, Rift Valley fever, and malaria surges.

## DISCUSSION

Recent outbreaks, epidemics, and pandemics of the viral hemorrhagic fevers (e.g., Ebola, Marburg), influenza, HIV, and Zika have taught us that communicable diseases can be only an airplane flight, a border, or a clinic away. Uganda has and continues to experience a number of these emerging and re-emerging infectious diseases, many occurring in epidemic proportions, due to its highly porous borders and its biogeographical location in an ecological hot spot and within several infectious disease transmission belts.^11^^,^^12^

To address these national and global health security challenges, Uganda set out to establish an NIPH to enable the country to better leverage, organize, and coordinate public health expertise and systems. The U.S. CDC—an NPHI in itself—together with the IANPHI and the Africa CDC have worked with African countries to strengthen an interconnected base of NPHIs throughout Africa. The goal is to form a network of accountable, efficient, proactive, and science-focused public health agencies that use data to drive decision making and contain global health threats at their source.^13^ Uganda has benefited tremendously from this assistance.

Uganda’s NIPH is part of a growing network of public health agencies in Africa that use data to drive decision making and contain global health threats at their source.

Uganda has followed 14 African countries in establishment of their NPHIs. Apart from the National Public Health Institute of Liberia, which only took 2 years to attain its legal status (2014–2016),^14^ many NPHIs have taken much longer to establish. For example, the Nigeria Centre for Disease Control took 12 years (2007–2019),^15^ the National Institute of Health Mozambique took 27 years (1991–2018),^16^ and other countries in Africa are still in the process of legally establishing their NPHIs. The lessons learned from these countries have informed the process of developing Uganda’s NIPH.

Uganda registered numerous successes in implementing the Global Health Security Agenda and scaling up IHR capacities to prevent, detect, and respond to numerous public health events, with the most recent being the 10^th^ EVD outbreak in the DRC^17^ and the COVID-19 pandemic. Binder et al. noted that donor funding rose in the recent past in support of immunization and HIV/AIDS but less rapidly for public health functions that are not direct services or linked to programs for high-priority diseases and conditions, most of which are housed in NPHIs. With more evidence that NPHIs are purveyors of public goods, the trend has changed. Uganda is a typical example of how donor support has helped deliver public goods to the population.^18^ The 2018 EVD outbreak in DRC, of which 2 imported cases spilled over to Uganda in 2019, was also controlled without transmission to the indigenous Ugandan community. These cases illustrate the coordinated and efficient system of the UNIPH.

Much of the impetus for the development of NPHIs in recent years has stemmed from the need to respond to global health security concerns. More recently, the emphasis on universal health coverage is highlighting the role NPHIs can play in achieving this vision.^19^ Several of the NPHI core functions articulated in the Africa CDC NPHI Framework, such as assessment of population health status and health promotion and prevention efforts, are critical to achieving Sustainable Development Goal 3, which calls for ensuring healthy lives and promoting well-being for all at all ages, and the specific subgoal related to health coverage.^20^ The importance of NPHIs both in ensuring health security, including rapidly identifying and responding to outbreaks and epidemics and in supporting the achievement of long-term population health goals, should help countries advocate for an increase of budgetary support for the public goods functions of NPHIs.

The recent emphasis on universal health coverage has highlighted the role NPHIs can play in achieving this vision.

### Lessons Learned

The Task Force and stakeholders identified the following best practices and key enablers for the successful establishment of the UNIPH.

- Having all UNIPH components adequately staffed enabled us to focus on the operational implementation of UNIPH components. All components except Public Health Research were staffed and functional.
- The Task Force brought various MOH programs on board, focusing on commonalities of functions (e.g., surveillance and data management for various disease programs were integrated through the Institute).
- The areas of public health emergencies and One Health were identified as strategic paths toward developing and galvanizing support for the UNIPH.
- RIA is critical for eliminating barriers to the legislative process.
- Effective and continuous communication enabled cooperation and collaboration with key institutions.
- Selection of competent leadership is crucial. An Interim UNIPH Director was appointed and worked closely with heads of the various components of UNIPH.
- Political buy-in was essential. All Ministers of Health and other political leaders in the MOH embraced the concept.

### Challenges

We encountered several challenges in the process of establishing the UNIPH: fear of the unknown by the incumbent MOH staff, who were unsure of their positions in the new organization; delayed buy-in from other government ministries and agencies; political factors due to the reshuffle of senior MOH leadership; and technical factors including retirement, turnover, and attrition of Task Force members. These challenges meant that several new teams or team members had to be introduced over time to the new concept of NPHIs, resulting in delays in the development process.

Another important challenge during this process was that the government banned the formation of new autonomous bodies and recommended mergers of some current ones to the mother ministries. However, by leveraging the resilience of the Minister of Health, the PHEOC, and the UNIPH interim leadership, as well as continuously highlighting the successes of the proposed UNIPH and advocating for its legalization in major meetings and presentations, the momentum was maintained, paving the way for the establishment of the UNIPH as an autonomous entity. Eventually, the explanation of the Minister of Health with clear justifications to the President of the Republic of Uganda was a success.

### Next Steps

To ensure consolidation of the various gains, the country is moving forward to complete the process of legalizing the status of its NIPH through an Act of Parliament. The architectural drawings of the UNIPH complex, which will house the Institute and its directorates near the National Health Laboratory buildings, have been completed and funding for construction of the structure is being actively sought.

The country is moving forward to complete the legalization of its NIPH through an Act of Parliament.

The next steps that will be undertaken to establish the UNIPH by an Act of Parliament are to finalize the consultation report (summary of all inputs from stakeholders) and obtain a certificate of financial implication (showing source of funding and how it will fit into government programs) from the Ministry of Finance, Planning, and Economic Development.

## CONCLUSION

We have documented the unique staged process used to develop the NIPH in Uganda. Strong political leadership, and stakeholder collaboration between the MOH and partners, and financial and technical support ensured the establishment of the UNIPH and streamlined its response to public health emergencies. The creation of an integrated disease control center enables better collaboration and synergies between different arms of epidemic preparedness and response.

## Notes

**Peer Reviewed**

**First published online:** July 27, 2022.
